# Mechanism of Dy^3+^ and Nd^3+^ Ions Electrochemical Coreduction with Ni^2+^, Co^2+^, and Fe^3+^ Ions in Chloride Melts

**DOI:** 10.3390/ma14237440

**Published:** 2021-12-04

**Authors:** Khasbi Kushkhov, Zhubagi Ali, Astemir Khotov, Anna Kholkina

**Affiliations:** 1 Department of Inorganic and Physical Chemistry, Kabardino-Balkarian State University, 360004 Nalchik, Russia; hasbikushchov@yahoo.com (K.K.); bagboshin@gmail.com (Z.A.); astemir.xotov@mail.ru (A.K.); 2 Institute of High Temperature Electrochemistry, Ural Branch, Russian Academy of Sciences, 620990 Ekaterinburg, Russia

**Keywords:** ionic melts, electrochemical coreduction, electrochemical synthesis, rare-earth intermetallic compounds, iron triad metals

## Abstract

The present paper is devoted to the study of the processes of the mechanism of electrochemical coreduction of Dy^3+^ and Nd^3+^ ions with Ni^2+^, Co^2+^, and Fe^3+^ ions in the equimolar NaCl-KCl melt at 973 K and characterization of the synthesized samples. The performed voltammetry analysis of the electrochemical coreduction processes elucidated a significant difference in the values of the extraction potentials of the studied metals. This melt testifies that intermetallic compounds of Dy and Nd with Ni, Co, and Fe may be synthesized in the kinetic regime. The intermetallic phases of Dy and Nd with Ni, Co, and Fe are found to be formed along with the phases of metallic Ni, Co, and Fe either during electrolysis at the cathode current densities exceeding the limiting diffusion current of Ni^2+^, Co^2+^, and Fe^3+^ ions or in the potentiostatic regime at the potentials of the corresponding voltammetry curves. Therefore, the following interrelated key parameters affecting the electrochemical synthesis of Dy and Nd intermetallic compounds with Ni, Co, and Fe were determined: (i) composition of the electrolyte, i.e., concentrations of FeCl_3_, CoCl_2_, NiCl_2_, DyCl_3_, and NdCl_3_; (ii) cathode current density or electrolysis potential and (iii) electrolysis time. The obtained samples were characterized by micro-X-ray diffraction analysis, cyclic voltammetry, and scanning electron microscopy methods.

## 1. Introduction

In the 21st century, emerging and conventional functional materials have a great impact on the quality of our lives. We have become technology-coddled, as technologies make our lives more comfortable. We are used to microwave ovens, laptops, digital phones, and high-speed transport. All these engineering achievements are possible because of new functional and construction materials. A special place should be given to the materials based on rare-earth intermetallic and refractory compounds, such as magnets, fluorescent substances, alloys for hydrogen accumulation, etc. Magnets, based on rare-earth metals (REM)—precisely, Nd-Fe-B—are becoming increasingly used, because of their record high characteristics as opposed to other permanent magnets. First, they exhibit higher magnetic fields and technical characteristics; secondly, these magnets are cheaper and more available due to the raw materials used for their fabrication. Apart from military–industrial complex and nuclear industry being traditional consumers of the novel materials, such fields as construction industry, oil and gas industry, chemical and metallurgical industries, medicine and microbiology, thermal energetics and machine building, wood working and food industries, radio electronics, and even outdoor advertisement are highly interested in new materials development.

Typical production processes for hard magnets, such as Nd-Fe-B, SmCo_5_, and others include either thermal reduction of Co and REM oxides by Ca or alloying of the preliminary obtained metals with the subsequent heavy grinding in inert atmosphere [1,2,3]. Among the drawbacks of these technological processes are their complexity, process temperatures exceeding 1000 °C, and the labor-consuming separation process of the end product from the formed slag that requires a reducing agent, i.e., metallic calcium obtained by molten salts electrolysis. Therefore, development of efficient production methods for REM-based hard magnetic materials are of supreme importance.

Molten salt electrolysis is one of perspective methods for the production of hard magnetic materials based on multicomponent rare-earth compounds. The processes of electrochemical co-deposition of the components of the synthesized compounds at the cathode and their further atomic interaction with the formation of nano-, submicro-, and microdisperse intermetallic and refractory powders lay the basis for the electrochemical synthesis of rare-earth intermetallic and refractory compounds [4]. Low energy consumption, simplified synthesis process and possibility to monitor and control the synthesis process, composition, and morphology of the end product are key advantages of the electrochemical synthesis method. However, there is a lack of literature data on the production of multicomponent REM compounds, especially Nd- and Dy-containing ones, via the electrodeposition from ionic melts.

The electrochemical synthesis of the intermetallic Ln_x_Me_y_ compounds (Ln = Dy, Nd; Me = Ni, Co, Fe) may be performed in three ways. The first one implies Nd and Dy electrodeposition from ionic melts on Ni, Co, or Fe (the iron triad) metallic substrates. In this case, the intermetallic compound is formed according to the reaction of Nd and Dy diffusion into the electrode bulk, which results in formation of diffusion coatings [5,6]. Thus, DyFe [7,8] Dy_2_Ni_3_ [9,10,11,12,13] intermetallic compounds were obtained in the form of diffusion coatings in molten KCl-LiCl containing Dy^3+^ ions. The alloy coatings of the DyNi_2_, DyCo_3_, and DyCo_2_ compositions were synthesized by the currentless diffusion alloying of the Ni and Co samples at 773–973 K in the molten KCl-LiCl eutectic containing DyCl_3_ [5]. In the second variant, soluble Fe, Ni, or Co consumable anodes serve as the sources of the iron triad ions. Edwar et al. obtained REM alloys with 3 d metals by the low-temperature electrolysis of molten salts containing Li and REM fluorides (sometimes with BaF_2_ additions) using soluble Fe and Co anodes [14]. The third variant implies the addition of the iron triad and REM chlorides to the molten alkali chloride background electrolyte. The electrochemical coreduction of REM ions as well as Ni^2+^, Co^2+^, and Fe^3+^ ions on the cathode and their further atomic interaction result in the formation of highly disperse intermetallic powder. Papers [15,16,17,18,19] reported that it is possible to synthesize intermetallic compounds containing Sa, La, Ho, and Ce with Co and Ni. Later, the processes of electrochemical coreduction of Dy^3+^ with Al^3+^ ions [20], Ho^3+^ with Al^3+^ [21], and Sm^3+^ with Ni^2+^ ions [22] were studied in the KCl-LiCl eutectic. These studies proved that Dy and Ho intermetallic compounds with Al and Sa with Ni can be electrochemically synthesized. Reviews [23,24,25] reveal the state-of-the-art achievements in the electrochemical synthesis of intermetallic and refractory compounds based on refractory and REM metals in chloride melts. A vast diversity of the synthesized materials and potential of this production method are promising for creation of new functional and constructive materials.

The present paper is aimed at the study of the processes of the electrochemical coreduction mechanism of Dy^3+^ and Nd^3+^ ions with ions of the iron triad in the KCl-NaCl eutectic. It is important to demonstrate the potential and to determine the mechanism of the electrochemical synthesis of intermetallic Ln_x_Me_y_ compounds (Ln = Dy, Nd; Me = Ni, Fe, Co).

## 2. Materials and Methods

### 2.1. Electrochemical Cell and Electrodes

The experiments were performed in the hermetically sealed, quartz, three-electrode cell in purified dry argon atmosphere. To eliminate the oxygen traces in the argon atmosphere, a zirconium pulp was put into the cell as a free oxygen getter. A glassy carbon crucible of 30 cm^3^ volume served simultaneously as a container for the melt and as an anode. A chloride–silver electrode Ag|KCl-NaCl (1:1)-AgCl (2.5 mol.%) was used as a reference electrode. A tungsten wire of 1.0 mm served as a cathode. An alundum tube dressed the tungsten wire leaving 40 mm uncovered. The area of the working electrode was calculated according to the immersion depth to the melt (10 ÷ 15 mm). The preparation and assembling of the cell were performed in the glove box mBraun Labstar 25 (MBRAUN, Garching, Germany) in pure argon atmosphere. To provide the operating temperature of 973 K, a shaft-type resistance furnace with silicon carbide heaters was used. The automatic regulation of the temperature was performed by an electronic regulator OVEN-TRM-1 with a chromel–alumel thermocouple, the accuracy of the temperature control was ±1 °C. Cyclic voltammograms and open-circuit potential measurements were obtained using an Autolab PGSTAT 30 device with IF-00 interface (Eco Chemie, Metrohm Autolab B.V., Utrecht, Netherlands). The voltammograms were processed using the software (GPES 4.9).

### 2.2. Methods for Analysis and Diagnostics of Cathode Deposits

To determine the phase and elemental composition of cathode deposits, an X-ray diffractometer D2 PHAZER (Bruker, Bremen, Germany); scanning electron microscope Tescan VEGA3LMH, which allows quantitative EDX chemical analysis (Tescan, Brno—Kohoutovice, Czech Republic); and X-ray fluorescent spectrometer Spektrscan MAKS GV (Spectron, St. Petersburg, Russia) were used.

### 2.3. Electrolyte Preparation

The KCl-NaCl equimolar melt was chosen as a background electrolyte. Chemically pure KCl and NaCl salts were preliminary dried in a vacuum oven for 10 h and then were annealed in an electric muffle for 5 h at 723 K. Dry chemically pure salts NdCl_3_, DyCl_3_, NiCl_2_, CoCl_2_, and FeCl_3_ (Chemcraft, Kaliningrad, Russia) were used as sources of Nd, Dy, and iron triad metals.

## 3. Results and Discussion

### 3.1. Electroreduction of Nd^3+^ and Dy^3+^ Ions in the KCl-NaCl Equimolar Melt at 973 K

We previously studied the electroreduction of the Nd*^3+^*and Dy*^3+^*ions in the KCl-NaCl melt on the active electrode materials (Ag, Pt, and GC) using DyCl_3_ and NdCl_3_ obtained via dewatering of NdCl_3_·6H_2_O and DyCl_3_·6H_2_O by dehydrating agents NH_4_Cl and CCl_4_ [26,27,28]. Experimental conditions and purity of the used reagents as well as the material of the electrode have a great impact on the structure, state, and electrochemical behavior of the Nd^3+^ and Dy^3+^ ions in chloride melts. In the present study, the electrochemical reductions of Nd^3+^ and Dy^3+^ ions with Ni^2+^, Co^2+^, and Fe^2+^ ions were performed in the equimolar KCl-NaCl melt using chemically pure reagents and inert electrode materials (W) at 973 K.

**Figure 1:**
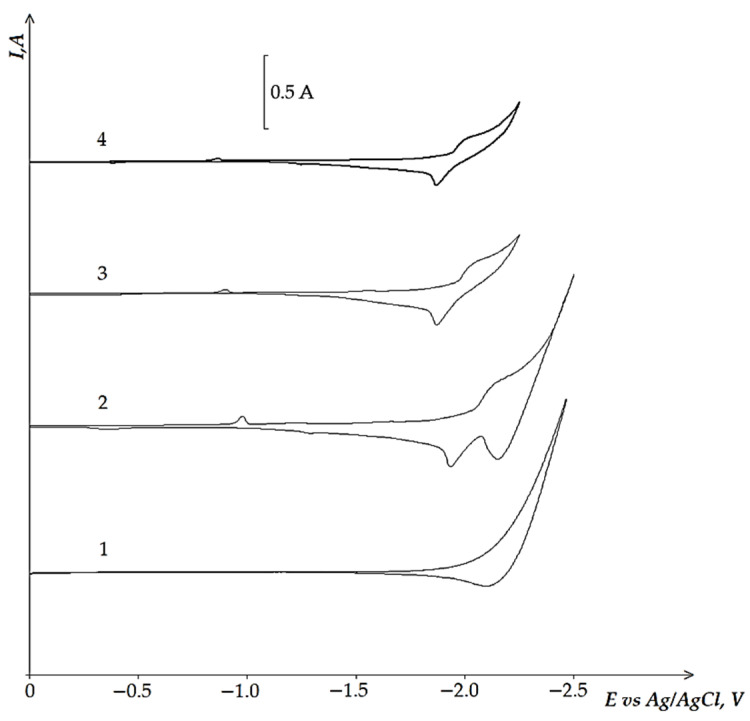
Cyclic voltammograms of the KCl-NaCl-NdCl_3_ melt on the tungsten electrode at 973 K and at different polarization rates: curves 1 and 2–0.2 V/s; curve 3–0.1 V/s; curve 4–0.05 V/s. Concentration of NdCl_3_: curve 1–0 mol/cm^3^; curves 2, 3, 4–4.0 × 10^−4^ mol/cm^3^. A square of the cathode is 0.42 cm^2^.

**Figure 2:**
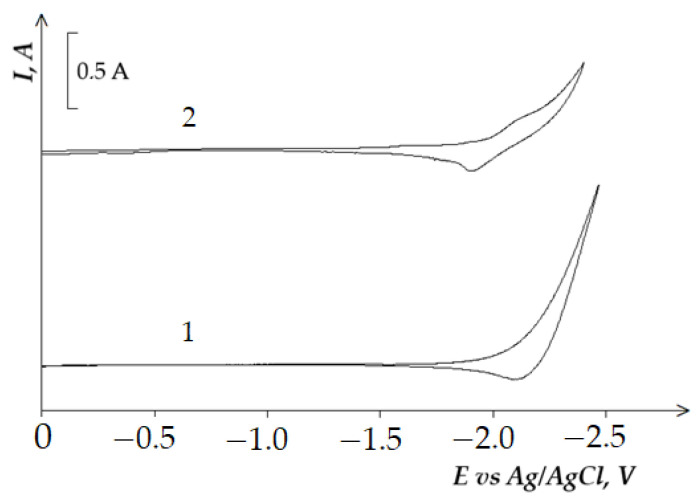
Cyclic voltammograms of the Dy^3+^ ions electroreduction on the tungsten electrode in the equimolar KCl-NaCl melt at 973 K. Concentration of DyCl_3_: curve 1–0 mol/cm^3^, curve 2–3.0 × 10^−4^ mol/cm^3^. A square of a cathode is 0.42 cm^2^. Polarization rate is 0.2 V/s.

Figure 1 presents cyclic voltammetry dependences of the equimolar KCl-NaCl melt containing 4.0 × 10^−4^ mol/cm^3^ of NdCl_3_ on the tungsten electrode at different polarization rates and at the temperature of 973 K. The absence of any other waves and low residual current of the voltammetry curve for the background KCl-NaCl electrolyte (Figure 1, curve 1) up to the potentials of the alkali metals extraction testifies to the high purity of the equimolar KCl-NaCl melt and approve its implementation in the proposed tasks. The addition of NdCl_3_ to the equimolar KCl-NaCl melt results in the appearance of the potential region −(1.85 ÷ 2.05) V relative to the Ag/AgCl reference electrode, which corresponds to the Nd^3+^ ions reduction. The height of this reduction wave increases as the polarization rate increases. The reduction waves of Nd^3+^ ions do not have a clear diffusion peak, because the Nd^3+^ reduction potentials are close to the potentials of the alkali metals extraction. The increase in the polarization rate and the transition to the non-stoichiometric regimes do not result in diffusion peak formation. However, the reduction wave was noticed to shift towards the region of negative potentials and to expand. The cyclic voltammetry dependencies of the equimolar KCl-NaCl melt containing DyCl_3_ (Figure 2) have only one wave of Dy^3+^ ions reduction in the potential region of −(1.9 ÷ 2.1) V and of the cathode product oxidation in the potential region of −(1.85 ÷ 2.05) V.

The analysis of the half-width of the Dy^3+^ and Nd^3+^ ions reduction waves according to the known diagnosis criteria [29,30] up to the polarization rates of 0.1 V/s elucidates that there are three electrons, which transfer during the cathode process. As the polarization rate increases, the half-width values of the Nd^3+^ and Dy^3+^ ions reduction waves increase. This testifies that the process of the Nd^3+^ and Dy^3+^ ions electrochemical reduction proceeds in the regime of controlled charge transfer rate.

Therefore, based on the structure of the chloride melts containing REM ions, the Nd^3+^ and Dy^3+^ ions were found to acquire the form of chloride LnCl_6_^3−^ complexes (Ln = Dy and Nd). The reversible three-electron reaction of the electrochemical cathode reduction has the following form:

LnCl_6_^3−^ + 3e ↔ Ln +6Cl^−^. (1)

### 3.2. Electrochemical Reduction of the Iron Triad Metals in the KCl-NaCl Melt at 973 K

The electrochemical reduction of Ni^2+^, Co^2+^, and Fe^3+^ ions in ionic melts is reported in a limited number of papers, because these processes are successfully performed in water solutions. However, chloride melts are the best media for production of powders and coatings from the iron triad metals. Thus, papers [31,32,33] report on the regularities of the Co^2+^ ions electrochemical reduction in the molten NaCl-CoCl_2_ and KCl-NaCl-CoCl_2_ systems. Sytchev et al. [32] studied the KCl-NaCl-FeCl_3_ (CoCl_2_, NiCl_2_) and KCl-NaCl-NaF(5 mol.%)-FeCl_3_ (CoCl_2_, NiCl_2_) melts by the cyclic voltammetry at 973 K. The character of the iron triad ions electrochemical reduction was determined and it was concluded that the electrode processes with the iron triad ions are diffusion-controlled. According to the research reported in [32], two electrons participate in the elementary processes of electrochemical reduction of Fe^3+^, Co^2+^, and Ni^2+^ both in chloride and in chloride–fluoride melts.

To eliminate the influence of the experimental conditions and impact of different electrode materials, we performed the electrochemical reduction of the iron triad ions under conditions similar to those of Dy^3+^ and Nd^3+^ ions’ electrochemical reduction.

**Figure 3:**
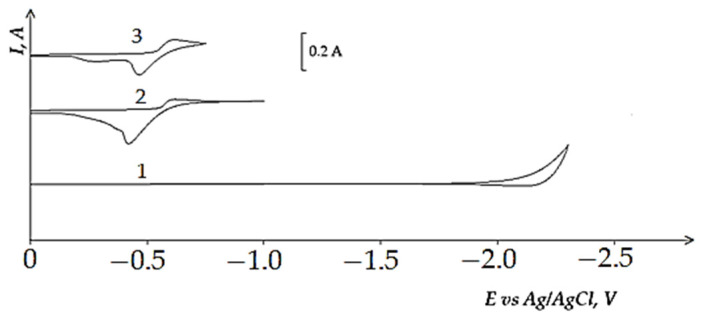
Cyclic voltammograms of the equimolar KCl-NaCl melt containing FeCl3, recorded on the tungsten electrode at 973 K. Concentration of FeCl3: curve 1–0 mol/cm^3^, curves 2 and 3–1.0 × 10^−4^ mol/cm^3^. Polarization rate is 0.2 V/s. A square of the cathode is 0.64 cm^2^.

**Figure 4:**
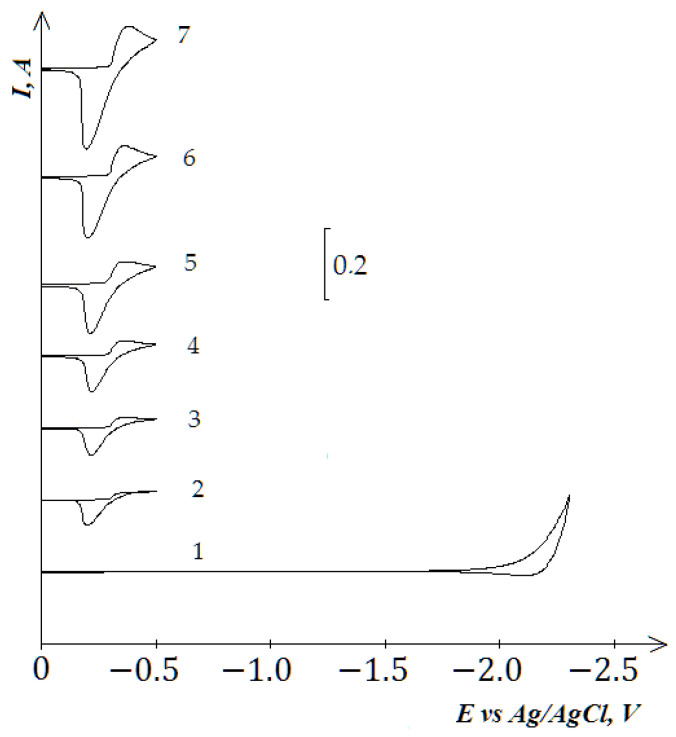
Cyclic voltammograms of the KCl-NaCl-CoCl_2_ melt recorded on the tungsten electrode at 973 K and at different polarization rates: curve 1–0.2 V/s, curve 2–0.02, curve 3–0.05 V/s, curve 4–0.1 V/s, curve 5–0.2 V/s, curve 6–0.5 V/s, curve 7–1.0 V/s. Curve (1) denotes the background KCl-NaCl (1:1) electrolyte; a square of the cathode is 0.64 cm^2^.

Figure 3 and Figure 4 present cyclic voltammograms recorded on the tungsten cathode during the electrochemical reduction of chloride Fe^3+^ and Co^2+^ complexes in the equimolar KCl-NaCl melt at 973 K. A wave of the Fe^3+^ and Co^2^ chloride complexes reduction is observed on the cathodic branch of cyclic voltammetry curves in the regions of potentials −(0.5 ÷ 0.6) V, −(0.3 ÷ 0.4) V relative to the Ag/AgCl reference electrode. The X-ray diffraction analysis of the product of potentiostatic electrolysis obtained at the potentials of the wave termination illustrates that the cathode deposit is composed on metallic Fe and Co phases. The anodic branch of the cyclic voltammogram has one wave of metallic Co oxidation. In the case of metallic Fe oxidation, two waves are observed. If the polarization reaches −1.0 V, the voltammetry curve stages of the metallic Fe electrochemical oxidation are less noticeable. The analysis of the Fe^3+^ ions reduction according to the diagnostic criteria of the chronopotentiometric method illustrates a three-electrode process of Fe^3+^ ions reduction, which is controlled by diffusion:

Fe^3+^ + 3e ↔ Fe^0^ (2)

The oxidation of metallic Fe proceeds in two stages:

Fe^0^ − 2e ↔ Fe^2+^ and Fe^2+^ − 1e ↔ Fe^3+^ (3)

The number of electrons (n), which transfer during the electrochemical reduction of Co^2+^ ions, was calculated using the value of the wave half-width for the polarization rates less than 0.1 V/s using equation E_p/2_ − E_p_ = 0.77RT/nF. It was found that n = 2.

Co^2+^ + 2e ↔ Co (4)

Electrochemical behavior of Ni^2+^ ions is analogous to that of Co^2+^ ions, with the only exception that nickel ions reduce in the more positive region of potentials −(0.05 ÷ 0.1) V.

### 3.3. Electrochemical Coreduction of Nd and Dy Ions with Iron Triad Ions in the KCl-NaCl Melt at 973 K

According to the phase diagrams of the Dy-Ni and Nd-Ni double metallic systems [34,35] Nd^3+^ and Dy^3+^ ions form the following intermetallic compounds with nickel Ln_2_Ni_17_, LnNi_5_, Ln_2_Ni_7_, LnNi_3_, LnNi_2_, LnNi, Ln_7_Ni_3_, Ln_3_Ni, where Ln = Dy and Nd. Nourry et al. calculated a free Gibbs energy of intermetallic compounds formation in the double system Nd_x_Ni_y_ and found that NdNi_5_ is the most stable phase (ΔG(NdNi_5_) = −350 kJ/mol) [36]. As the Nd^3+^ concentration increases in the intermetallic compounds, the free Gibbs energy increases but its value does not exceed zero at 1073 K (ΔG(Nd_3_Ni) = −110 kJ/mol). Among all analyzed phases, only NdNi_5_, NdNi, DyNi_5_, and DyNi are considered as congruently melting compounds. The rest are incongruently melting compounds that form according to the peritectic reaction. In the double Dy-Co system, there are two congruently melting compounds—Dy_2_Co_17_, DyCo_3_—and five incongruently melting ones—Dy_2_Co_7_, DyCo_2_, DyCo_3_, Dy_12_Co_7_, Dy_3_Co. The Nd-Co system has one congruently melting compound—Nd_2_Co_17_—and nine incongruently melting ones—NdCo_5_, Nd_2_Co_7_, Nd_5_Co_19_, NdCo_3_, NdCo_2_, Nd_2_Co_3_, Nd_2_Co_1.7_, Nd_2_Co_17_, Nd_7_Co_3_, Nd_3_Co. The phase diagram of the Dy-Fe system illustrates the formation of two congruently melting compounds Dy_2_Fe_17_ and DyFe_3_ and two incongruently melting compounds Dy_6_Fe_23_ и DyFe_2_. The phase diagram of the Nd-Fe system demonstrates the formation of only two incongruently melting compounds, Nd_2_Fe_17_ and Nd_5_Fe_17_ [34,35]. According to these data we may estimate that the electrochemical synthesis of dysprosium and neodymium intermetallic compounds with nickel and cobalt is more feasible than those with iron.

To determine the possibility of electrochemical coreduction of Nd^3+^ and Dy^3+^ ions with the iron triad ions, we performed voltammetry measurements of the equimolar KCl-NaCl melt containing Nd^3+^, Dy^3+^, Fe^3+^, Co^2+^, and Ni^3+^ ions. The experiments and electrochemical measurements were performed in three different ways:

(i) First, measured portions of the NiCl_2_, CoCl_2_, and FeCl_3_ were added to the equimolar melt and the voltammetry dependencies of the electrochemical reduction process were recorded. Then, NdCl_3_ and DyCl_3_ were added to the iron triad containing KCl-NaCl melt and the voltammetry dependence was recorded considering their bulk concentration in the background electrolyte.
(ii) NdCl_3_ and DyCl_3_ were first added to the equimolar KCl-NaCl melt, the voltammetry dependence of the molten KCl-NaCl-NdCl_3_ (DyCl_3_) system was recorded; then, iron triad ions were added to the previously formed mixture and the voltammograms of electrochemical coreduction were recorded.
(iii) The calculated amount of the background electrolyte compounds KCl and NaCl (1:1), NdCl_3_ and DyCl_3_, as well as NiCl_2_, CoCl_2_, and FeCl_3_ were mixed in a dry powder form, put into a glassy carbon crucible and melted in the three-electrode cell in argon atmosphere of a dry box with continuously increasing temperature up to 973 K.

The experiments demonstrated that the sequence of iron triad and REM chlorides addition to the equimolar mixture does not affect the behavior of the voltammetry dependences of the electrochemical coreduction.

**Figure 5:**
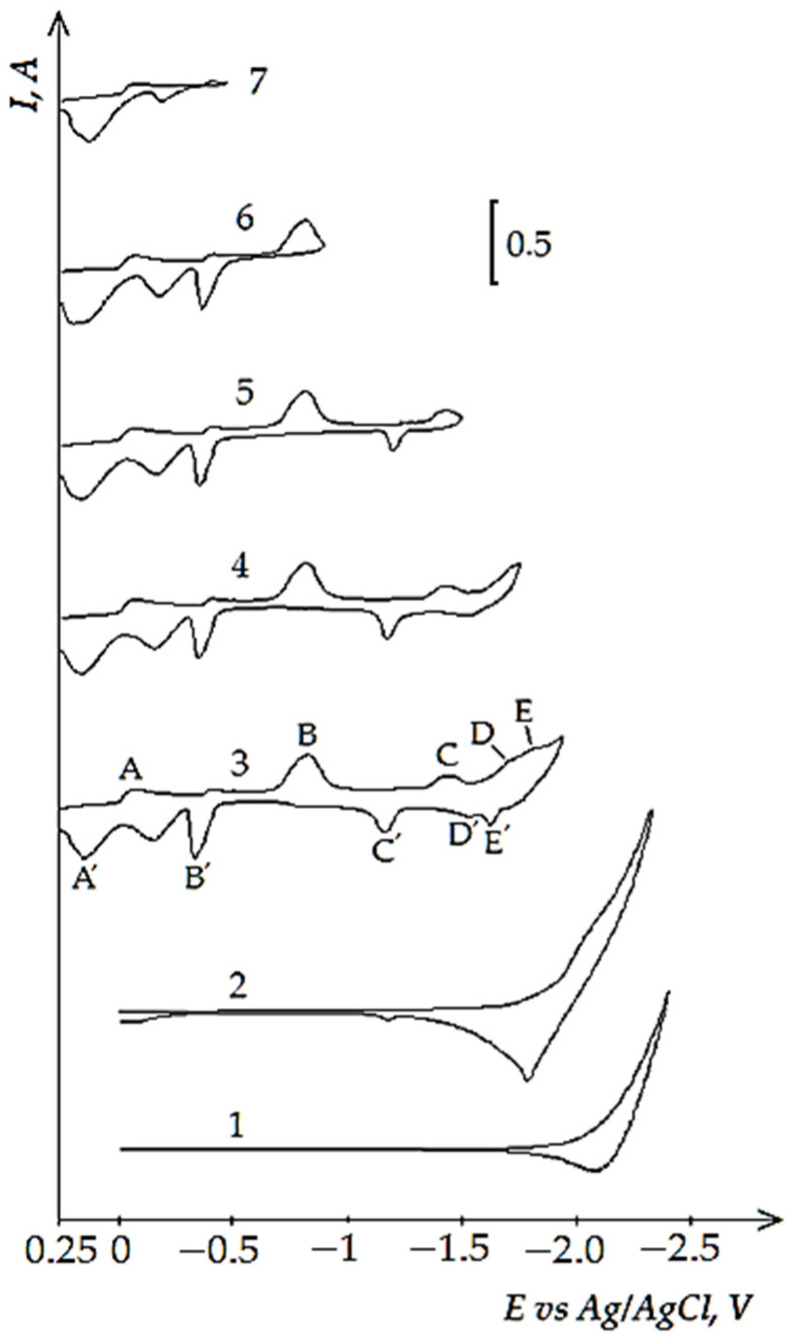
Cyclic voltammograms of electrochemical coeducation of Nd^3+^ and Ni^2+^ ions on the tungsten electrode in the equimolar KCl-NaCl melt at 973 K, polarization rate of 0.2 V/s at different reverse potentials: curves (1 and 2)—(−2.4 V), curve 3 (−1.94 V), curve 4 (−1.75 V), curve 5 (−1.5 V), curve 6 (−0.9 V), curve 7 (−0.48 V). Concentrations of NdCl_3_: curve 1—0 mol/cm^3^; curves (2–7)—4.0 × 10^−5^ mol/cm^3^. Concentrations of NiCl_2_: curves (1 and 2)—0 mol/cm^3^, curves (3–7)—1.0 × 10^−4^ mol/cm^3^. A square of the cathode is 0.4 cm^2^.

**Figure 6:**
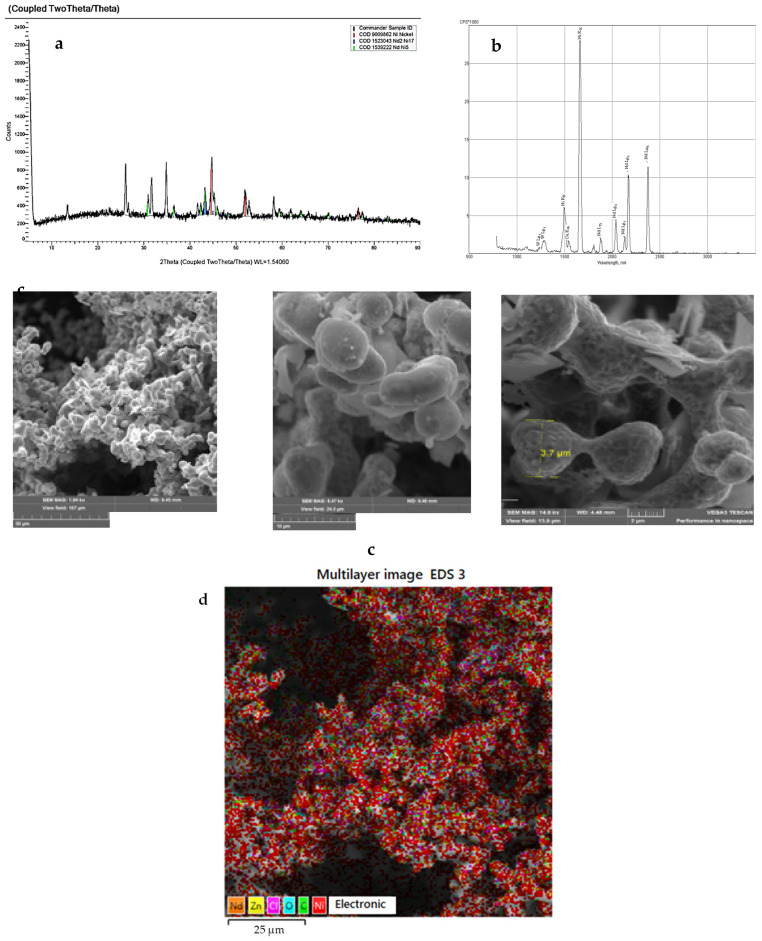
X-ray diffraction pattern (**a**), elemental analysis (**b**), SEM images of the synthesized samples surfaces (**c**), and color map of the elemental distribution (**d**) of the cathode deposit obtained by the potentiostatic electrolysis of the KCl-NaCl-NdCl_3_-NiCl_2_ melt at the voltage of 3.2 V, electrolysis potential of −1.5 V vs. Ag/AgCl, and at 973 K for 5 h. Concentration of NdCl_3_ is 3.0 × 10^−4^ mol/cm^3^. Concentration of NiCl_2_ is 0.5 × 10^−4^ mol/cm^3^.

Figure 5 presents cyclic voltammograms of the process of electrochemical coreduction of NdCl_3_ and NiCl_2_. The addition of NdCl_3_ to the KCl-NaCl-NiCl_2_ chloride melt changes the pattern of voltammetry curves and results in the appearance of several extra reduction waves apart from the Ni^2+^ ions reduction waves at the potentials of −(0.0 ÷ 0.1) V (wave A). The reduction wave B was recorded at the potentials of −(0.7 ÷ 0.8) V, wave C appeared at the potentials of −(1.6 ÷ 1.7) V, wave D at the potentials of −(1.6 ÷ 1.7) V, and wave E at the potentials of −(1.9 ÷ 2.0) V (see Figure 5, curve 3). Wave E corresponds to the process of pure metallic Nd extraction (see Figure 2, curve 2). The appearance of the waves B, C, and D on the voltammogram is related to the reduction of Nd^3+^ ions with depolarization onto the preliminarily extracted metallic Ni on the tungsten cathode. These waves illustrate the formation of the intermetallic Nd_x_Ni_y_ phases of various compositions. Thus, according to the X-ray diffraction analysis, the cathode deposit after 5 h of potentiostatic electrolysis at the potentials of the wave B termination (−1.0 V) is mainly composed of preliminarily deposited metallic Ni phase and small amount of the Nd_2_Ni_17_ intermetallic phase. The obtained X-ray diffraction pattern (Figure 6a) demonstrates that the cathode deposit at the potentials of wave C (−1.5 V) is composed of the mixture of metallic Ni and intermetallic Nd_2_Ni_17_ and NdNi_5_ phases and, according to the elemental analysis illustrated in Figure 6b, the electrolysis product contains mainly Ni and Nd elements. The obtained scanning electron microscopy (SEM) images illustrate microfine particles of elongated and round forms (Figure 6c). Their location at the surface of the studied sample is defined using the color mapping. The corresponding color map provided in Figure 6d testifies that intermetallic Nd_x_Ni_y_ phases are present in the analyzed sample. The cathode deposit at the termination of wave D is composed of metallic Ni, intermetallic NdNi_5_ and NdNi_2_ phases, and traces of the Nd_2_Ni_17_ phase. In addition, the more negative electrolysis potential results in higher Nd concentration in the intermetallic compounds. The anodic branch of the cyclic voltammogram also has 5 waves: A’, B’, C’, D’, and E’. The correspondence of the cathodic reduction waves and anodic electrochemical oxidation waves of the cathode cycle are verified by the voltammetry curves recorded before the reverse potentials, corresponding to the termination of each cathodic reduction wave (Figure 5, curves 4–7).

**Figure 7:**
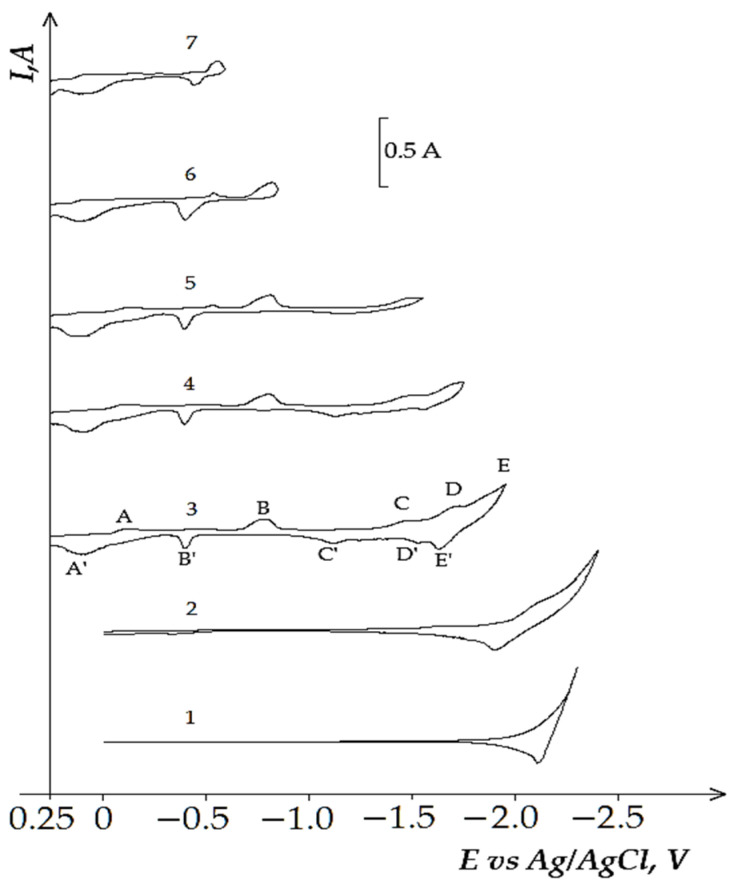
Cyclic voltammograms of electrochemical coreduction of Dy^3+^ and Ni^2+^ ions on the tungsten electrode in the equimolar KCl-NaCl melt at 973 K, polarization rate of 0.2 V/s, and different reverse potentials: curve 1 (−2.3 V), curve 2 (−2.4 V), curve 3 (−2.0 V), curve 4 (−1.75 V), curve 5 (−1.76 V), curve 6 (−0.8 V), curve 7 (−0.6 V). Concentrations of DyCl_3_: curve 1—0 mol/cm^3^, curves (2–7)—3.0 × 10^−4^ mol/cm^3^. Concentrations of NiCl_2_: curves (1 and 2)—0, curves (3–7)—5.0 × 10^−5^ mol/cm^3^. A square of the cathode is 0.4 cm^2^.

**Figure 8:**
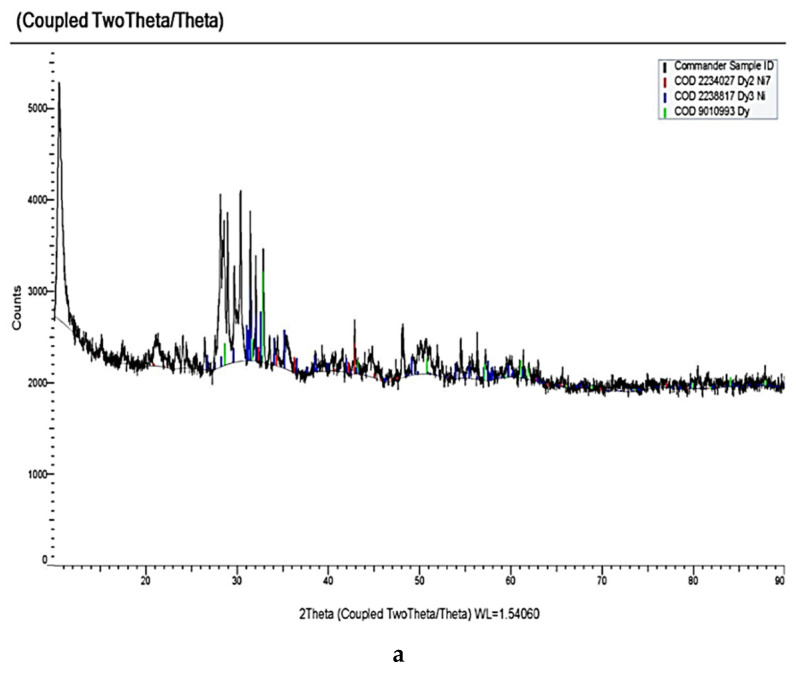

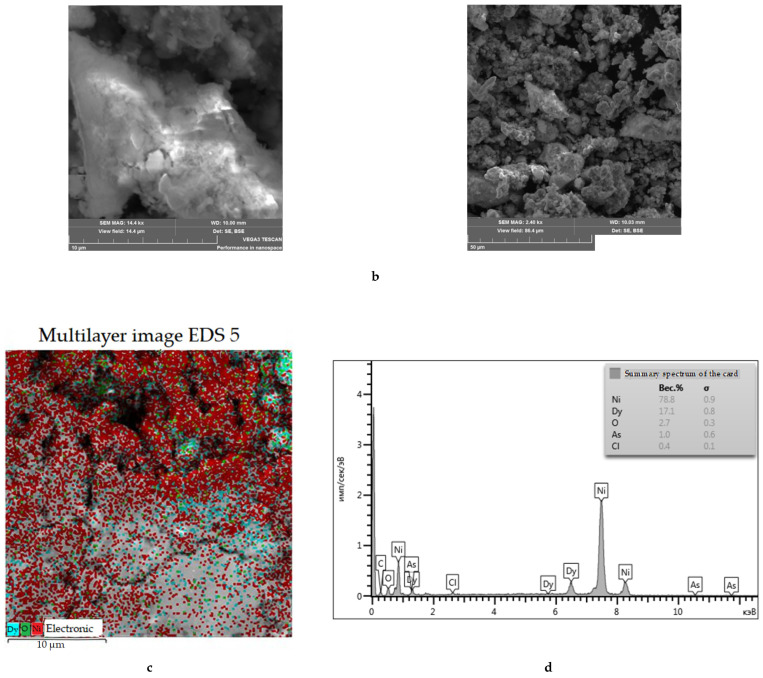
X-ray pattern (**a**), SEM images of the surface (**b**), color map of elemental distribution (**c**), and elemental analysis (**d**) of the cathode deposit obtained by electrolysis of the KCl-NaCl-DyCl_3_-NiCl_2_ melt at 973 K for 2 h, at the voltage of 3.4 V, and electrolysis potential of −1.6 V vs. Ag/AgCl. Concentration of DyCl_3_ is 3.0 × 10^−4^ mol/cm^3^. Concentration of NiCl_2_ is 0.5 × 10^−4^ mol/cm^3^.

The analogous changes in the voltammograms are observed in the equimolar KCl-NaCl melt containing DyCl_3_ and NiCl_2_. Thus, the voltammetry dependence of the KCl- NaCl-DyCl_3_-NiCl_2_ melt (Figure 7) illustrates five reduction waves: wave A illustrates Ni^2+^ ions reduction V on the tungsten electrode at the potentials of −(0.0–0.1); waves B, C, and D are associated with the reduction of Dy^3+^ ions with the depolarization of the preliminarily extracted metallic Ni on the tungsten cathode, which results in the formation of various intermetallic compounds—Dy_2_Ni_17_, DyNi_5_, and DyNi_2_; wave E denotes the extraction of pure Dy on the intermetallic compounds. The anodic part of the cyclic voltammogram also illustrates five waves: A’, B’, C’, D’, and E’. Their correspondence to the cathodic reduction waves is verified by the voltammograms until the moment when the values of the reverse potentials become different, which corresponds to the termination of each cathodic reduction wave (Figure 7, curves 3–7). This suggestion is exemplified by the X-ray pattern of the obtained cathode deposits illustrated in Figure 8a, microphotographs of the surfaces of synthesized samples (Figure 8b), color map of the elemental distribution (Figure 8c), and their elemental analysis (Figure 8d). The cathode deposits were synthesized by electrolysis of the equimolar KCl-NaCl-DyCl_3_ (3.0 × 10^−4^ mol/cm^3^)-NiCl2 (0.5 × 10^−4^ mol/cm^3^) melt for 2 h at 973 K and at the potentials corresponding to the wave C termination (−1.6 V vs. Ag/AgCl) and electrolytic cell voltage of 3.4 V. SEM images of the synthesized powder elucidate the formation of agglomerates composed of metallic Ni microparticles and DyNi_5_ intermetallic compound. The X-ray pattern obtained by the X-ray diffraction microanalysis demonstrates the presence of Dy and Ni elements in the cathode deposit. The formation of the Dy-Ni phase is also observed at the color map of elements distribution.

Based on the aforesaid, the electrochemical processes during electrolysis of molten KCl-NaCl-NdCl_3_ (DyCl_3_)-NiCl_2_ systems are described as follows:

wave A → Ni^2+^ + 2e = Ni (5)

wave B 2 NdCl_6_^3−^(DyCl_6_^3−^) + 6e + 17Ni = Nd_2_(Dy)_2_Ni_17_ +12Cl^−^ (6)

wave C NdCl_6_^3−^(DyCl_6_^3−^) + 3e + 5Ni = Nd(Dy)Ni_5_ +6Cl^−^ (7)

wave D NdCl_6_^3−^(DyCl_6_^3−^) + 3e + 2Ni = Nd(Dy)Ni_2_ +6Cl^−^ (8)

wave E NdCl_6_^3−^(DyCl_6_^3−^) + 3e = Nd(Dy) + 6Cl^−^ (9)

The process illustrated by wave D proceeds on the Nd(Dy)Ni_5_ intermetallic compound and is accompanied with a certain depolarization. Extracted metallic Nd and Dy interact with Nd(Dy)Ni_5_ and form phases with large Nd and Dy content.

2Nd(Dy) Ni_5_ + 3Nd(Dy) = 5Nd(Dy) Ni_2_ (10)

**Figure 9:**
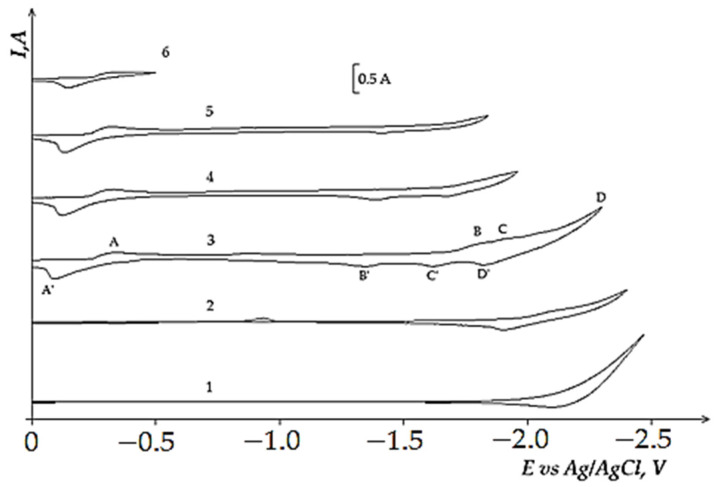
Cyclic voltammograms of electrochemical coreduction of Dy^3+^ and Co^2+^ ions on the tungsten electrode in the equimolar KCl-NaCl melt at 973 K, polarization rate of 0.2 V/s, and different reverse potentials: curves (1 and 2)—(−2.4 V), curve 3 (−2.1 V), curve 4 (−1.96 V), curve 5 (−1.84 V), curve 6 (−0.4 V). Concentrations of DyCl_3_: curve 1—0 mol/cm^3^, curves (2–6)—3.0 × 10^−4^ mol/cm^3^. Concentrations of CoCl_2_: curve (1 and 2)—0 mol/cm^3^, curves (3–6)—5.0 × 10^−5^ mol/cm^3^. A square of the cathode is 0.4 cm^2^.

**Figure 10:**
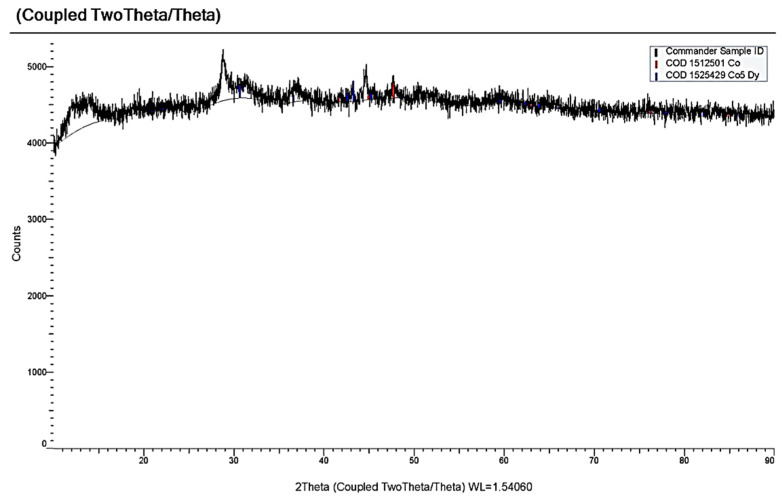
X-ray pattern of the cathode deposit obtained by electrolysis of the KCl-NaCl-DyCl_3_-CoCl_2_ melt at 973 K for 1 h and the voltage of 3.4 V. Concentration of DyCl_3_ is 3.0 × 10^−4^ mol/cm^3^. Concentration of CoCl_2_ is 0.5 × 10^−4^ mol/cm^3^.

Figure 9 illustrates the voltammograms of the electrochemical coreduction process of DyCl_3_ and CoCl_2_. The addition of CoCl_2_ to the molten KCl-NaCl-DyCl_3_ (3.0 × 10^−4^ mol/cm^3^) system results in the appearance of the cathodic part of the voltammetry dependence, apart from the DyCl_6_^3−^ ions reduction wave (Figure 9, curve 3, wave D) and other three waves. Wave A appears at the potentials of –(0.2 ÷ 0.25) V, wave B at −(1.6 ÷ 1.7) V, and wave C at −(1.8 ÷ 1.9) V. The electrochemical reduction of Co^2+^ ions to metallic cobalt is illustrated by wave A. The appearance of waves B and C on the voltammograms is associated with the dysprosium ions on the preliminarily extracted metallic Co on the tungsten electrode. The formation of intermetallic Dy_x_Co_y_ phases of various compositions is illustrated by these waves, as in the case of the chloride KCl-NaCl-DyCl_3_ melt with NiCl_2_ concentration. The X-ray diffraction analysis of the cathode deposit obtained by the potentiostatic electrolysis at the potentials of the wave B termination (−1.7 V) performed during 5 h illustrated that the cathode deposit is mainly composed of the metallic Co phase and small amounts of DyCo_5_ intermetallic compound (Figure 10). The electrolysis product at −1.7 V is composed of the metallic Co phase and intermetallic DyCo_5_ and DyCo_2_ phases. In addition, the more negative electrolysis potential results in higher Dy concentration in the intermetallic compounds. The anodic branch of the cyclic voltammogram has four waves (A’, B’, C’, D’) corresponding to the anode dissolution of metallic Co (A’), metallic Dy (D’), and intermetallic phases (B’ and C’). The anodic waves correspond to the cathodic reduction waves. This fact is verified by the voltammetry curves recorded before the potentials, corresponding to the termination of each cathodic reduction wave (Figure 9, curves 3–6).

**Figure 11:**
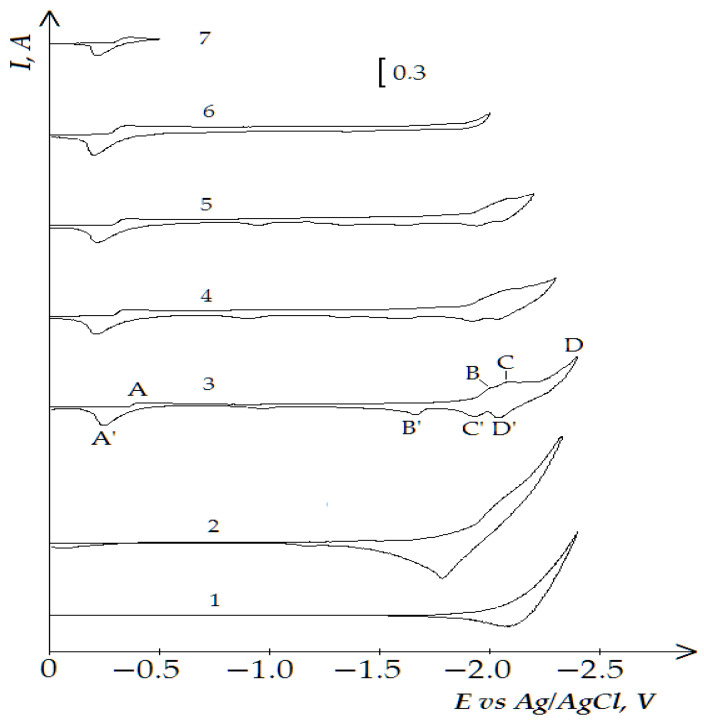
Cyclic voltammograms of electrolytic coreduction of neodymium and cobalt ions on the tungsten electrode in the equimolar KCl-NaCl melt at 973 K, polarization rate of 0.2 V/s, and different reverse potentials: curve (1 and 2)—(−2.3 V), curve 3 (−2.4 V), curve 4 (−2.3 V), curve 5 (−2.2 V), curve 6 (−2.0 V), curve 7 (−0.5 V). Concentrations of NdCl_3_: curve 1—0 mol/cm^3^, curves (2–7)—3.0 × 10^−4^ mol/cm^3^. Concentrations of CoCl_2_: curves (1 and 2)—0 mol/cm^3^, curves (3–7)—5.0 × 10^−5^ mol/cm^3^. A square of the cathode is 0.4 cm^2^.

**Figure 12:**
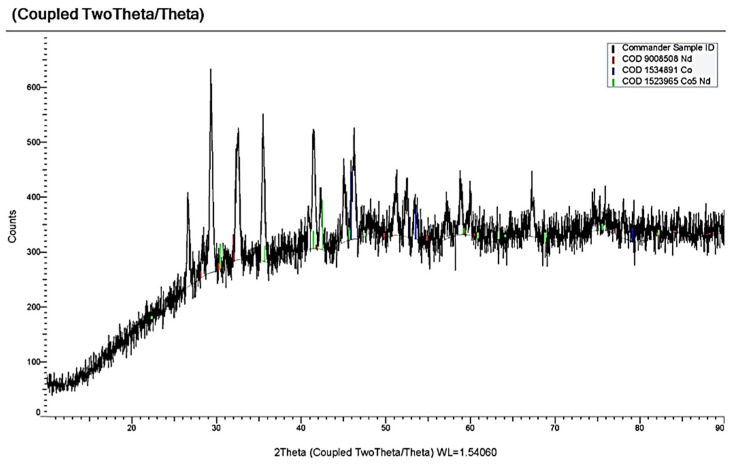
X-ray pattern of the cathode deposit obtained by electrolysis of the KCl-NaCl-NdCl_3_-CoCl_2_ melt at 973 K for 1 h and the cell voltage of 3.4 V. Concentration of NdCl_3_ is 3.0 × 10^−4^ mol/cm^3^. Concentration of CoCl_2_ is 0.5 × 10^−4^ mol/cm^3^.

Figure 11 demonstrates four waves on the dependencies of the molten KCl-NaCl-NdCl_3_-CoCl_2_ system until the potential of the background electrolyte is reached: wave A appears at the potentials −(0.3 ÷ 0.4) V, an extended wave at the potentials −(1.9 ÷ 2.2) V, which tends to half halving on wave B at −(1.9 ÷ 2.1) V and wave C at −(2.1÷ 2.2) V (Figure 11). Wave A denotes the electrochemical reduction of Co^2+^ ions to metallic Co (see Figure 4). Waves B and C illustrate the electroreduction accompanied with depolarization of NdCl_6_^3-^ complexes on the preliminary extracted metallic cobalt with further formation intermetallic neodymium and cobalt compounds NdCo_5,_ NdCo_2_ (Figure 12), respectively. Wave D is associated with the electrochemical extraction of metallic Nd on the previously reduced Nd_x_Co_y_ intermetallic compound. The anodic branch presents the electrochemical oxidation of the cathodic waves’ products. Besides, the anodic branch provides a more precise half halving of the cathodic waves B and C oxidation (waves B’ and C’).

Given the obtained data, the electrochemical processes that take place during the molten KCl-NaCl-NdCl_3_ (DyCl_3_)-CoCl_2_ salts electrolysis are presented as follows:

wave B NdCl_6_^3−^(DyCl_6_^3−^) + 3e + 5 Co = Nd(Dy) Co_5_ + 6Cl^−^ (11)

wave C NdCl_6_^3−^(DyCl_6_^3−^) + 3e + 2Co = Nd(Dy)Co_2_ + 6Cl^−^ (12)

**Figure 13:**
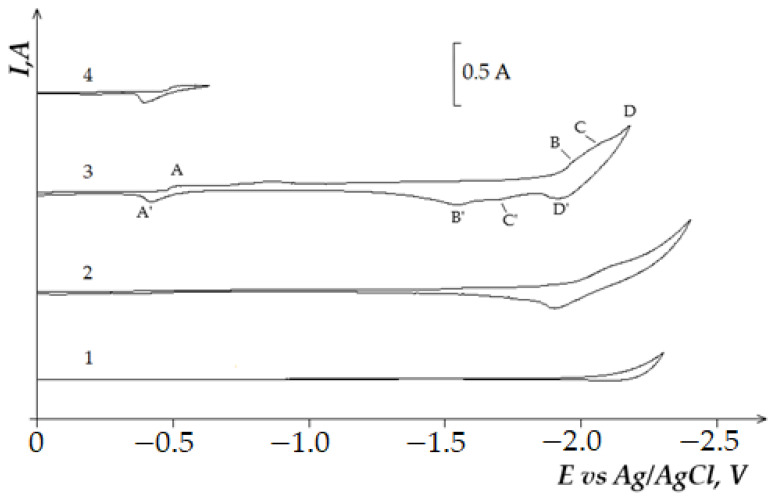
Cyclic voltammograms of electrochemical coreduction of Dy^3+^ and Fe^3+^ ions on the tungsten electrode in the equimolar KCl-NaCl melt at 973 K, polarization rate of 0.2 V/s, and different reverse potentials: curve 1 (−2.3 V), curve 2 (−2.4 V), curve 3 (−2.2 V), curve 4 (−0.62 V). Concentration of DyCl_3_: curve 1—0 mol/cm^3^, curves (2–4)—3.0 × 10^−4^ mol/cm^3^. Concentration of FeCl_3_: curves (1 and 2)—0 mol/cm^3^, curves (3 and 4)—5.0 × 10^−5^ mol/cm^3^.

The addition of FeCl_3_ to the KCl-NaCl-DyCl_3_ melt results in the appearance of the well-reproducible wave of the Fe^3+^ ions reduction at the potentials −(0.5–0.6). This process is accompanied with the extraction of the phase of metallic Fe^3+^ phase (Figure 13, curves 3 and 4).

**Figure 14:**
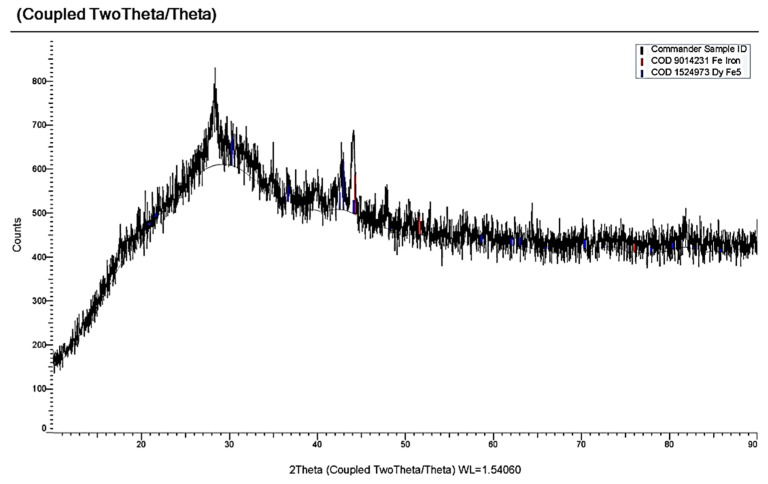
X-ray pattern of the cathode deposit obtained by electrolysis of the KCl-NaCl-DyCl_3_-FeCl_3_ melt at 973 K for 1 h and the cell voltage of 2.8 V. Concentration of DyCl_3_ is 3.0 × 10^−4^ mol/cm^3^. Concentration of FeCl_3_ is 1.5 × 10^−4^ mol/cm^3^. Electrochemical cell voltage = 2.8 V; T = 973 K; t = 1 h.

The wave of dysprosium chloride DyCl_6_^3−^ complex’s extraction shifts insignificantly to the region of more positive potentials −(1.8–2.0) V. On the cathodic branch of the voltammetry dependence of the KCl-NaCl-DyCl_3_-FeCl_3_ melt, a clear wave of DyCl_6_^3−^ reduction that may be associated with the formation of Dy_x_Fe_y_ intermetallic phases is not observed, as opposed to that of the melt containing CoCl_2_ and NiCl_2_. The anodic branch of the cyclic voltammogram has four waves related to the dissolution of the metallic Fe (A’) and Dy (D’) as well as the waves corresponding to the dissolution of the Dy_x_Fe_y_ intermetallic phases (B’ and C’). Figure 14 presents the X-ray pattern of the cathode deposit synthesized via electrolysis of the KCl-NaCl-DyCl_3_-FeCl_3_ melt at 973 K and electrolytic cell voltage of 2.8 V.

**Figure 15:**
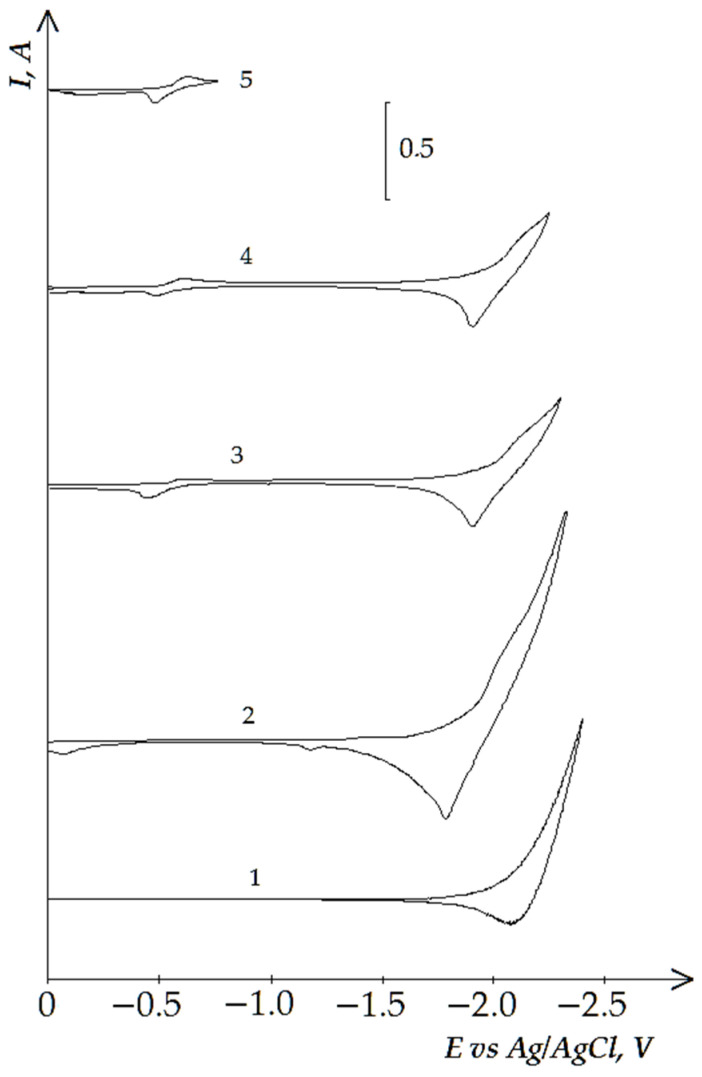
Cyclic voltammograms of electrochemical coreduction of Nd^3+^ and Fe^2+^ ions on the tungsten electrode in the equimolar KCl-NaCl melt at 973 K and polarization rate of 0.2 V/s recorded at different reverse potentials: curves 1 and 2 (−2.4 V), curve 3 (−2.23 V), curve 4 (−2.25 V), curve 5 (−0.75 V). Concentrations of NdCl_3_: curve 1—0 mol/cm^3^, curves (2–5)—4.0 × 10^−4^ mol/cm^3^. Concentrations of FeCl_3_: curves (1 and 2)—0 mol/cm^3^, curves (3–5)—5.0 × 10^−5^ mol/cm^3^. A square of the cathode is 0.64 cm^2^.

**Figure 16:**
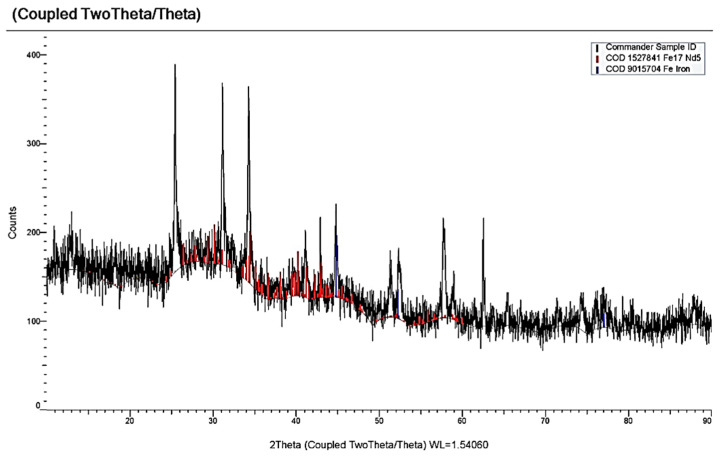
X-ray pattern of the cathode deposit obtained by electrolysis of the KCl-NaCl-NdCl_3_-FeCl_3_ melt at 973 K for 1 h and the cell voltage of 2.8 V. Concentration of NdCl_3_ is 4.0 × 10^−4^ mol/cm^3^. Concentration of FeCl_3_ is 1.0 × 10^−4^ mol/cm^3^.

The addition of NdCl_3_ to the KCl-NaCl-FeCl_3_ melt results in the formation of Nd^3+^ ions at potentials of −(2.0–2.2) V (Figure 15). The main peculiarity of the NdCl_6_^3−^ complex’s reduction in the KCl-NaCl-FeCl_3_ melt is that the Nd^3+^ ions extraction on the tungsten electrode coated with deposited metallic Fe is accompanied by small depolarization, caused by the formation of Nd_x_Fe_y_ intermetallic compounds (Figure 16), as opposed to the similar process performed in the KCl-NaCl melt (Figure 1).

The comparative analysis of the voltammograms of the KCl-NaCl-DyCl_3_ (NdCl_3_)- MeCl_x_ (where Me = Ni, Co, Fe) melts illustrates that among the iron triad, Ni most actively interacts with metallic Dy and Nd; this interaction results in the formation of intermetallic La_x_Ni_y_ compounds (La = Nd and Dy). Co is the second most active metal of the iron triad, whereas Fe is the least active one. A similar comparison of Dy and Nd illustrates that Dy interacts more actively with the iron triad than Nd. Apart from that, a significant difference in the potentials of iron triad and Dy(Nd) extraction (~1.2 ÷ 1.5 V) testifies that the electrolytic synthesis of intermetallic Ln_x_Me_y_ compounds (Ln = Dy, Nd; Me = Ni, Fe, Co) is possible only in the kinetic regime.

## 4. Conclusions

Based on the voltammetry studies of the electrochemical coreduction of Dy^3+^, Nd^3+^, Ni^2+^, Co^2+^, and Fe^3+^ ions in the equimolar KCl-NaCl melt at 973 K, it is shown that the electrochemical synthesis of these intermetallic compounds may be performed only in the kinetic regime. It is determined that the interaction activity resulting in the formation of intermetallic compounds between metallic Dy and Nd with the iron triad decreases in a row, starting with Ni, Co, and Fe; whereas, Dy is found to be more active in the aforesaid interactions than Nd.

It is shown that depending on the electrolysis regime (galvanostatic and potentiostatic), the process of the electrochemical synthesis consists of the following subsequent stages. It is found that only metallic Ni, Co, and Fe phases are extracted on the cathode during the galvanostatic electrolysis at cathode current densities smaller than the density of the limiting diffusion current of the iron triad ions electroreduction at the potentiostatic electrolysis up to the potentials of the limiting current of nickel, cobalt, and iron ions’ electrochemical reduction. The phases of intermetallic Ln_x_Me_y_ compounds (Ln = Dy, Nd; Me = Ni, Fe, Co) were detected together with the metallic Ni, Fe, and Co phases on the voltammograms recorded during electrolysis at the cathode current density exceeding the limiting diffusion current of Ni^2+^, Co^2+^, and Fe^3+^ ions or during potentiostatic electrolysis at the potentials of the corresponding waves.

In addition, the higher cathode current density and more negative electrolysis potentials result in the smaller concentration of iron triad metals in the cathode deposit and higher concentration of the intermetallic phases, including intermetallic phases with great REM concentration. For this reason, the process of electrochemical synthesis of intermetallic compounds Ln_x_Me_y_ compounds (Ln = Dy, Nd; Me = Ni, Fe, Co) is determined by the following interrelated parameters: composition of the electrolyte (concentrations of FeCl_3_, CoCl_2_, NiCl_2_, DyCl_3_, and NdCl_3_), cathode current density of the electrolysis potential (electrochemical cell voltage), and electrolysis time. Further, we plan to perfect these regimes and to select the optimal parameters for the electrochemical synthesis, diagnose the samples, and determine the magnetic field characteristics.

## Data Availability

The data presented in this study are contained within the article.
